# Silencing of directional migration in *roundabout4 *knockdown endothelial cells

**DOI:** 10.1186/1471-2121-9-61

**Published:** 2008-11-03

**Authors:** Sukhbir Kaur, Ganesh V Samant, Kallal Pramanik, Philip W Loscombe, Michael L Pendrak, David D Roberts, Ramani Ramchandran

**Affiliations:** 1 Department of Pediatrics, Children's Research Institute, Medical College of Wisconsin, Milwaukee, WI, USA; 2 Genome Technology Branch, National Human Genome Research Institute, National Institutes of Health, Bethesda, MD, USA; 3 University of Scranton, Biology Department, Scranton, PN, USA; 4 Laboratory of Pathology, National Cancer Institute, National Institutes of Health, Bethesda, MD, USA

## Abstract

**Background:**

Roundabouts are axon guidance molecules that have recently been identified to play a role in vascular guidance as well. In this study, we have investigated gene knockdown analysis of endothelial Robos, in particular *roundabout 4 *(*robo4*), the predominant Robo in endothelial cells using small interfering RNA technology *in vitro*.

**Results:**

*Robo1 and Robo4 *knockdown cells display distinct activity in endothelial cell migration assay. The knockdown of *robo4 *abrogated the chemotactic response of endothelial cells to serum but enhanced a chemokinetic response to Slit2, while *robo1 *knockdown cells do not display chemotactic response to serum or VEGF. *Robo4 *knockdown endothelial cells unexpectedly show up regulation of Rho GTPases. Zebrafish Robo4 rescues both Rho GTPase homeostasis and serum reduced chemotaxis in *robo4 *knockdown cells. Robo1 and Robo4 interact and share molecules such as Slit2, Mena and Vilse, a Cdc42-GAP. In addition, this study mechanistically implicates IRSp53 in the signaling nexus between activated Cdc42 and Mena, both of which have previously been shown to be involved with Robo4 signaling in endothelial cells.

**Conclusion:**

This study identifies specific components of the Robo signaling apparatus that work together to guide directional migration of endothelial cells.

## Background

Major classes of axon guidance molecules include the Netrins, Semaphorins, Ephrins and Slit ligands, which interact with their cognate family of receptors to orchestrate stereotypical nerve patterns in a developing vertebrate embryo [1]. Each family has at least one member that plays a functional role in vascular development. Our study focuses on the Roundabout (Robo) family of axon guidance genes [2]. Robos are cell surface transmembrane receptors that have been identified in most species to mediate repulsion-guidance mechanisms in axons [3]. Four robo receptor genes (*robo1-4*) have been identified in mammals, and their function vary widely depending on the tissue where they are expressed [4]. Robo4, the fourth member of the Robo family is expressed in both the neural and vascular systems [5,6]. Robo4 knockdown zebrafish embryos display intersomitic vessel (ISVs) sprouting defects [5]. More recently, *robo4*^*AP/AP *^knockout mice show defects in vascular integrity that exacerbates pathological conditions associated with vascular leakage [7].

Robo1 and Robo4 are the two endothelial relevant Robos. Robo1 has been functionally implicated in the vasculature; in the context of tumor growth [8]. Robo1 and Robo4 share similar domains in both extracellular and intracellular cytoplasmic regions, but they differ widely in the number and spatial organization of these domains [9]. Robo1 contains five-immunoglobulin (IgG) and three-fibronectin domains compared to three and two respectively for zebrafish Robo4 [9]. Human Robo4 differs slightly from zebrafish Robo4 in that it only contains two IgG and two fibronectin domains [10]. In the intracellular region, Robo1 and Robo4 share two of the four conserved cytoplasmic (CC) motifs CC0 and CC2. Robos are known to homo- and hetero-dimerize, and dimerization is responsible for mediating signal transduction in neurons [11].

Slits are ligands for Robos [12-14]. Slit2 has been implicated as the vascular-specific Slit and has been studied extensively [10,15-17]. Contradictory results are reported in the literature regarding Slit2's role in migration of endothelial cells. Two groups report that Slit2 inhibits migration of endothelial cells [6,18] and other groups report that Slit2 mediates positive stimulus on endothelial cells [8,19]. Axon guidance molecules are well known to show such dual function [1]. For example, Slits were originally identified as attractants of sensory axons [13] and were later identified as repellents for Robo^+ ^axons [12,20].

To date, the functional output of Robos in endothelial cells is unresolved [10]. Some reports indicate that Slit2 binds to Robo1 in endothelial cells and promotes migration of these cells [8,19]. However, in neurons, Slit-Robo1 interaction primarily mediates repulsive signals [12,21]. In the case of Robo4, the Slit2-Robo4 interaction is implicated to inhibit migration of endothelial cells *in vitro *[6,18]. We have demonstrated that Robo4 also induces positive signal in endothelial cells and have implicated attraction mechanisms [22]. Interestingly, soluble Robo4 shows anti-angiogenic effects *in vitro *supporting for this possibility [23].

To facilitate clarity with Robos' function in endothelial cells *in vitro*, we have initiated a loss-of-function study of Robos' *in vitro*. To investigate the loss of function phenotype for *robo1 *and *robo4 *in vitro, we have used Dicer siRNA technology to knock down endogenous *robo *transcripts in endothelial cells. *Robo1 *and *Robo4 *siRNAs specifically knocks down the respective Robo RNAs and proteins in endothelial cells without affecting the other. *Robo4 *siRNA knockdown endothelial cells did not respond to serum but *Robo1 *siRNA knockdown cells do. Slit2 binds to endothelial cells expressing both Robos but does not bind to *robo4 *knockdown endothelial cells. Interestingly, *robo4 *siRNA cells display pro-migratory response to Slit2 despite lack of binding of Slit2. This suggests an alternative migration induced by Slit2 in the absence of Robo4. Indeed, Slit2 displays chemotactic and chemokinetic activity on *robo4 *knockdown endothelial cells. Serum, on the other hand displays an exclusive chemotactic activity on endothelial cells, which is abolished in the absence of Robo4.

We had previously implicated Rho GTPases in Robo4-induced migration signaling complex in endothelial cells [22]. Here, we show that *robo4 *knockdown endothelial cells contain increased Rho GTPase level, which is restored by transfecting back zebrafish Robo4 suggesting that any perturbation of Robo4 levels on endothelial cell surface results in alteration of Rho GTPase homeostasis. Importantly, this restoration is critical for endothelial cell chemotaxis to serum. Slit2 treatment of endothelial cells does not induce Rho GTPases suggesting that the mechanism of Slit2 inhibition of endothelial cell migration is Rho GTPase independent.

To explain the mechanism used by Robos for directing endothelial cell migration, we have identified that Robo1 and Robo4 interact with each other and share several molecules. They both bind to Slit2, Mena [6,24] and Vilse, a Cdc42-GAP. In addition, we implicate IRSp53, a Cdc42 target that is down-regulated in *robo4 *knockdown cells that show no organized actin stress fiber arrangements. Our data suggests that a complex of proteins (Cdc42-GTP, IRSp53, Mena, Vilse) are part of a serum mediated Robo4 signaling axis that is responsible for directional migration of endothelial cells.

## Results and discussion

### *Robo4 *siRNA specifically knocks down *robo4 *RNA and protein

**Figure 1:**
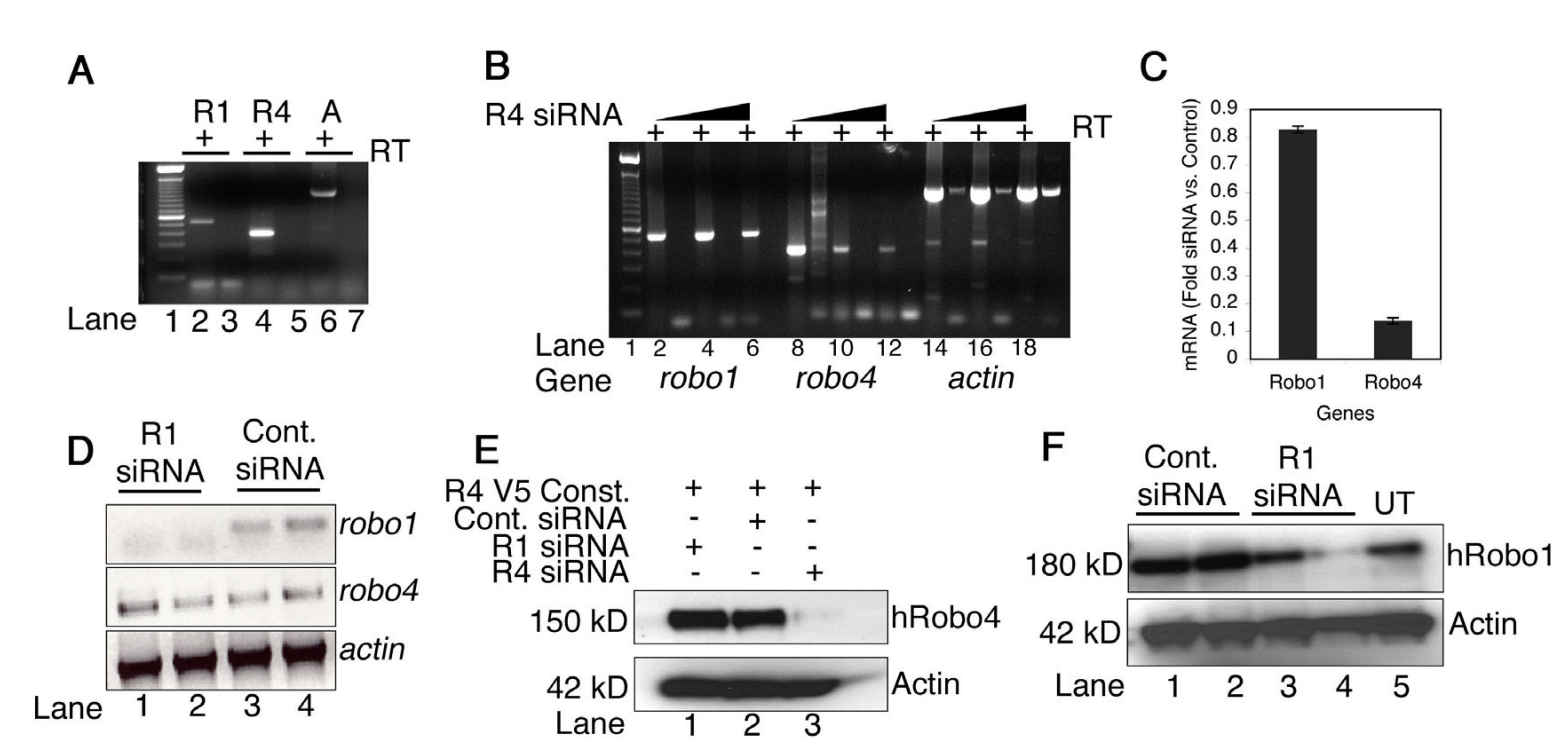
**siRNA mediated knockdown of *robo4 *and *robo1***. **A **shows RT-PCR gel for *robo1 *(R1), *robo4 *(R4) and *actin *(A) transcripts in the presence (+) of reverse transcriptase (RT) from HUVEC total RNA. The relative expression level of *robo1 *has percentile rank of 79 ± 4.5% and *robo4 *is slightly higher at 83.7 ± 5.7% in HUVECs [35,36]. Numbers on the bottom of gel represent lane numbers. **B **depicts RT-PCR for *robo1*, *robo4 *and *actin *genes in the presence (+) of reverse transcriptase (RT) from total RNA isolated from HUVECs transfected with increasing concentrations (bars) of *robo4 *siRNA. For *robo1 *gene: lanes 4 and 6, *robo4 *gene: lanes 10 and 12, and *actin *gene: lanes 16 and 18 represent 125 and 250 ng of siRNA respectively. Lanes 2, 8 and 14 are RNA isolated from untransfected HUVECs, and represent endogenous levels of each transcript. **C **indicates the ratios of *robo1 *and *robo4 *transcripts by real time PCR in control *lacZ *siRNA and *robo4 *siRNA transfected cells. **D **shows the RT-PCR for *robo1*, *robo4*, and *actin *transcripts in *robo1 *siRNA (lanes 1 and 2) or control *lacZ *(lanes 3 and 4) siRNA cells. **E **shows western blots for V5-human Robo4 and actin protein in 293 cell lysates from samples transfected with respective siRNA indicated by +. **F **shows western blot for endogenous Robo1 and Actin protein levels in control *lacZ *siRNA (Cont. siRNA) or *robo1 *(R1 siRNA) and untransfected (UT) 293 cells. Lanes 1 and 3, 2 and 4 are 250 and 500 ng of respective siRNAs indicated on the top of the gel. Lanes 15, 17 and 19 have spillover of excess actin product in -RT lanes.

We have first determined the expression levels of endothelial Robos in human umbilical vein endothelial cells (HUVECs). HUVECs show robust expression of *robo4 *when compared to *robo1 *(compare +RT lanes R4 with R1 in Fig. 1A). To knockdown endogenous *robo4*, we have utilized the RNA interference technology [25] that relies on Dicer enzyme, which converts double stranded RNA into small interfering RNA (siRNA). We used the Dicer method of generating pooled *robo4 *small interfering RNA (siRNA) for a Robo4 region that was distinct from other Robos since it is difficult to predict *a priori *which region of Robo4 is optimal for targeting using siRNA. Increasing concentrations (125–500 ng) of *robo4 *siRNA resulted in a dose dependant reduction of *robo4 *transcript (Fig. 1B, lanes 8–13) with minimal change in *robo1 *(Fig. 1B, lanes 2–7) or *actin *transcript levels (Fig. 1B, lanes 14–19). Quantitative real time PCR analysis showed that *robo4 *transcript is down regulated by 80% when compared to *robo1 *in *robo4 *siRNA cells (Fig. 1C). Using the Dicer method, we also designed *robo1 *siRNA and show that *robo1 *siRNA selectively targets *robo1 *transcript (Fig. 1D, compare lanes 1–2 to 3–4) with no effect on *robo4 *RNA levels (Fig. 1D, *robo4 *gel).

To investigate whether *robo4 *RNA knockdown correlates with protein levels, we generated a V5-tagged human Robo4 fusion construct and co-transfected it with control *lacZ *(Fig. 1E, lane 2) or *robo4 *siRNA (Fig. 1E, lane 3) in 293T cells. To unambiguously show that Robo4 protein was down in *robo4 *siRNA cells, we took the transfected V5-tagged approach. Protein lysates from *robo4 *siRNA sample show lower amounts (Fig. 1E, lane 3, hRobo4 gel) of V5-tagged protein when compared to control *lacZ *siRNA co-transfected sample (Fig. 1E, lane 2, hRobo4 gel) or *robo1 *siRNA sample (Fig. 1E, lane 1, hRobo4 gel). Similarly *robo1 *siRNA also shows selective knockdown of endogenous Robo1 protein (Fig. 1F, lanes 3 and 4, hRobo1 gel) with no effect on transfected V5-tag Robo4 protein (Fig. 1E, lane 2) when compared to control *lacZ *siRNA cells (Fig. 1F, lanes 1 and 2, hRobo1) or untransfected (UT) cells (Fig. 1F, lane 5, hRobo1 gel). Taken together, these results suggest that we have generated specific knockdown reagents that can selectively target Robo1 and Robo4 in endothelial cells.

### Serum-mediated migration responses are selectively abrogated in *robo4 *knockdown endothelial cells

**Figure 2:**
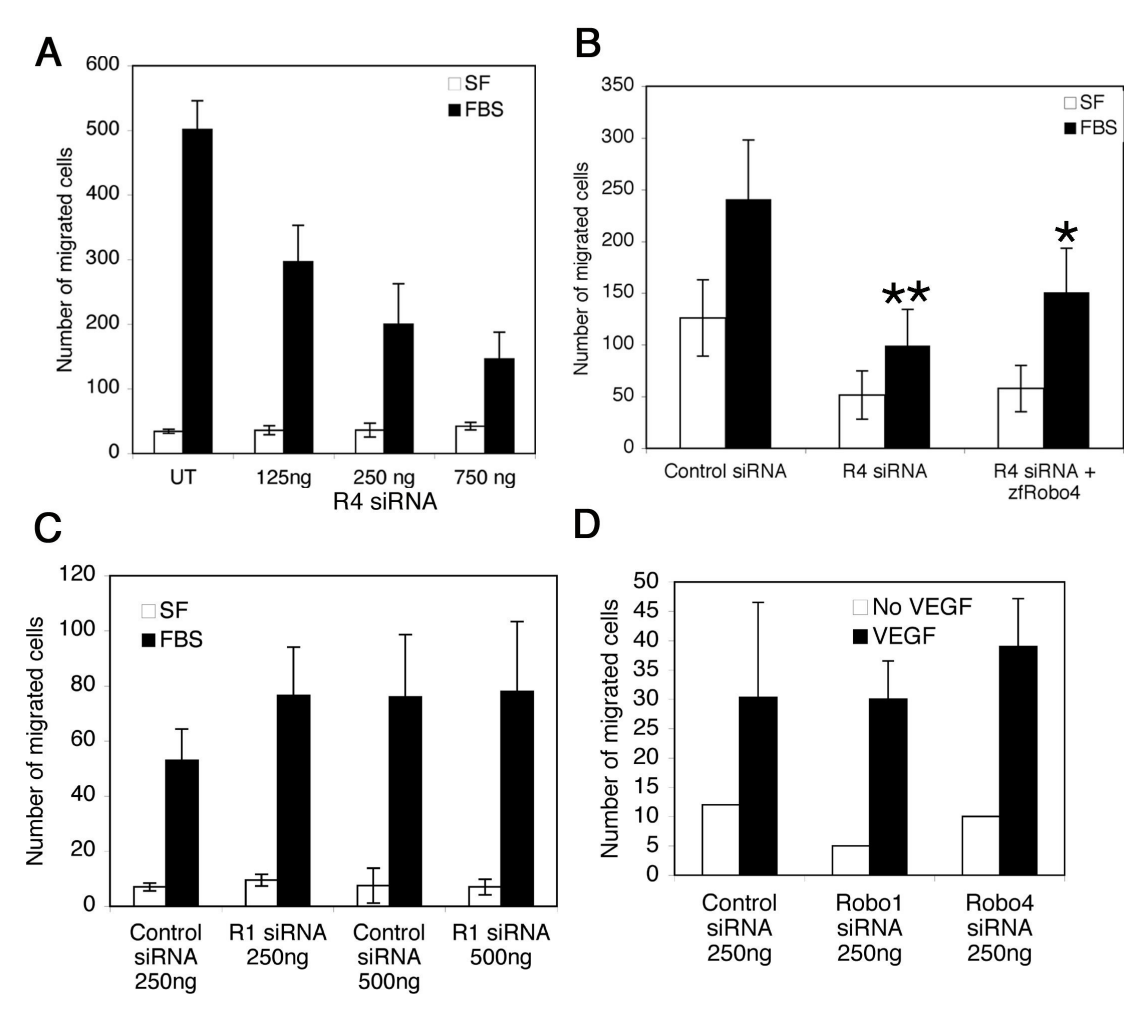
***Robo4 *knockdown endothelial cells do not migrate to serum**. **A **shows the migration assay for HUVECs transfected with 125–750 ng of siRNA in response to fetal bovine serum (FBS, black bar) or serum free (SF) conditions. **B **shows migration assay for HUVEC transfected with control *lacZ *siRNA (125 ng), *robo4 *siRNA (125 ng), *robo4 *siRNA (125 ng) plus zebrafish Robo4 (2 μg) (zfRobo4) constructs in response to fetal bovine serum (FBS) or serum free conditions (SF). Results were derived from three independent experiments (n = 3) with triplicate samples each time and the data is expressed as mean +/- SD. Two group comparisons were determined by Student two-sample *t *test assuming equal variances, and statistical significance was considered for *p *< 0.05. ** *p *< 9.83e-06 for control *lacZ *siRNA and R4 siRNA, and *p < 0.003 for R4 siRNA and R4 siRNA plus zfRobo4 samples. **C **shows the migration assay with control *lacZ *or *robo1 *siRNA transfected endothelial cells with the amounts indicated on the *x*-axis to fetal bovine serum (FBS) or serum free conditions (SF). This experiment was repeated twice with triplicate sample each time. **D **shows migration assay with control *lacZ *or *robo1 *or *robo4 *siRNA transfected endothelial cells to VEGF (100 ng/mL). This experiment was repeated three times with duplicate sample each time. Error bars in C and D represent SD across three independent experiments. In panels A-D, FBS was added to the bottom well.

To investigate the *robo4 *knockdown phenotype in endothelial cells, we have performed modified Boyden chamber migration assays in the presence of serum in the bottom chamber using endothelial cells transfected with *robo4 *siRNA (125–750 ng) (Fig. 2A) or control *lacZ *siRNA (Fig. 2B). Wild type untransfected HUVECs showed a robust response to serum (Fig. 2A, compare UT black and open bars), and cells transfected with increasing concentrations of *robo4 *siRNA showed a dose dependent decrease in migration (Fig. 2A, compare black bars 125–750 ng). No difference in apoptosis was noted between control *lacZ *and *robo4 *siRNA transfected cells by Annexin-FITC assay (data not shown). Co-transfecting a zebrafish Robo4 construct into *robo4 *siRNA transfected cells rescued the migration response to serum (Fig. 2B, R4siRNA + zfRobo4), suggesting that loss of migration phenotype was indeed specific to Robo4. We have also performed migration experiments with *robo1 *siRNA endothelial cells to serum and observed no change in response (Fig. 2C) when compared to *lacZ *siRNA cells. To determine growth factor mediated responses to migration, we have performed migration assay with vascular endothelial growth factor (VEGF-A) on *lacZ*, *robo1*, and *robo4 *siRNA endothelial cells and found no difference in migration across the three samples (Fig. 2D). These results argue that a specific stimulus (VEGF-independent) in serum triggers Robo4 but not Robo1 mediated directional migration.

The loss of migration phenotype in *robo4 *knockdown endothelial cells implies that Robo1, the other endothelial Robo cannot compensate for Robo4 function in endothelial cells. We also have already shown previously [22] that the phenotype induced by Robo1 and Robo4 on endothelial cells are different. Robo1 induces actin fibers that are long and thin while Robo4 induces short and thick actin bundles along with membrane ruffles. Therefore, it is possible that Robo1 and Robo4 serve different functions in endothelial cells.

### Slit2 binds to human endothelial cells

**Figure 3:**
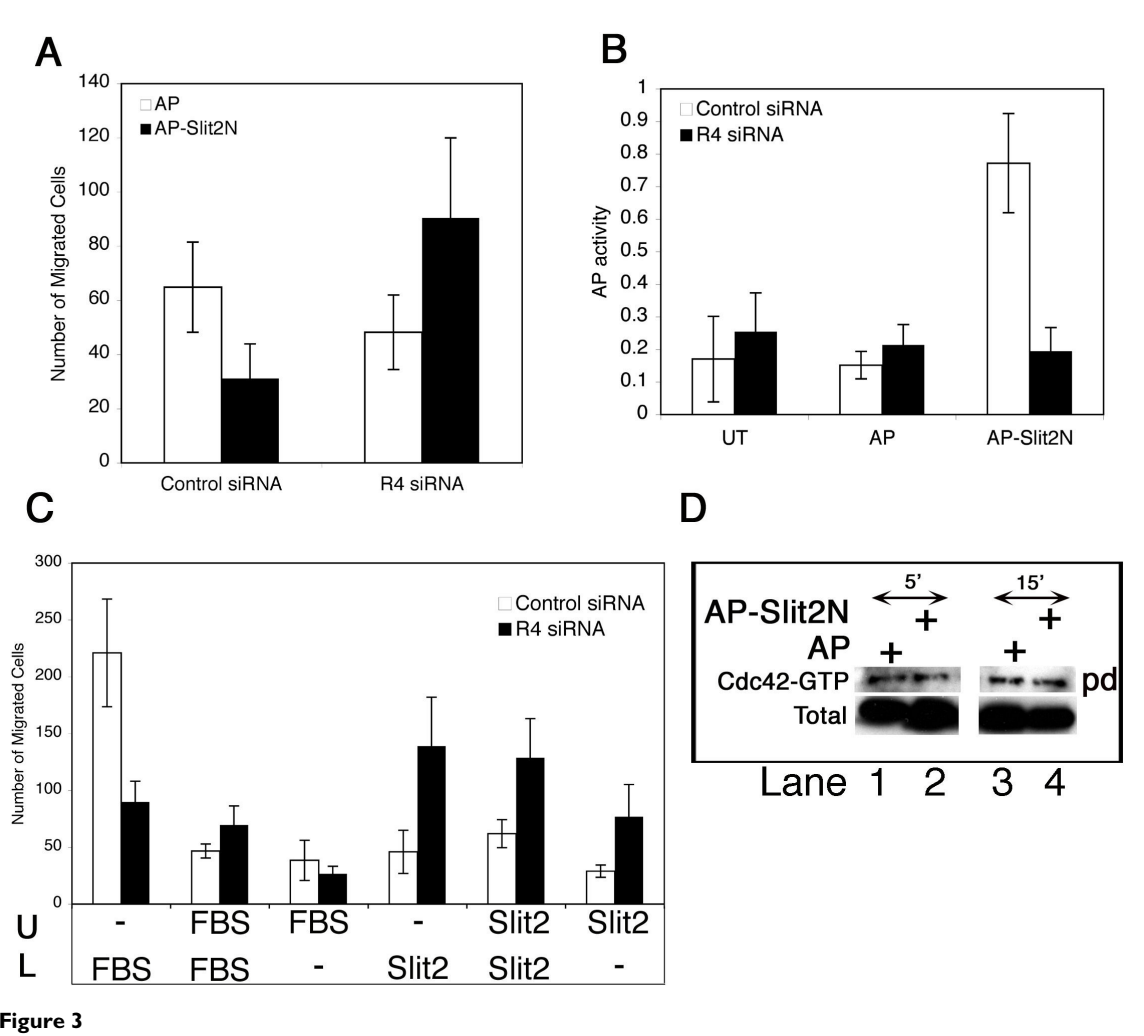
**Slit2 mediates chemokinetic and chemotactic behaviour on endothelial cells while serum exclusively mediates chemotaxis**. **A **shows the migration of control *lacZ *siRNA and *robo4 *siRNA transfected endothelial cells to AP and AP-Slit2N (25 ng/ml) fusion proteins in a Boyden chamber assay. The data here is consolidated from three independent experiments with each experiment performed with samples in triplicate. **B **shows the AP activity in lysates prepared from untransfected (UT), AP and AP-Slit2N treated control and *robo4 *siRNA transfected endothelial cells. **C **shows migration assay for control *lacZ *and *robo4 *siRNA transfected cells to Serum or AP-Slit2N in either upper (U), lower (L) or both chambers as indicated. Error bars in **A **(n = 3), and **B **(n = 3) represent SD while in **C **represent SEM (n = 4). **D **shows pulldown analysis of Cdc42-GTP levels in AP and AP-Slit2N (25 ng/ml) treated endothelial cell lysates for 5 and 15 minute respectively. + indicate addition of the reagent on the left, pd: pulldown, total: total Cdc42 protein in lysates.

Human Slit2 has been previously suggested to be a ligand for Robo4 [6,18], and is proteolytically processed into 140 kDa N-terminal and 55–60 kDa C-terminal fragments in both cell culture and *in vivo *[13,14]. We have performed migration assay with alkaline phosphatase (AP)-tagged N-terminal fragment of Slit2 (AP-Slit2N), which in the bottom chamber had previously been shown to be the active fragment of full-length Slit2 [13]. Migration of control *lacZ *siRNA transfected cells is inhibited by AP-Slit2N while migration of *robo4 *siRNA cells is not (Fig. 3A). To determine if Slit2 binding is altered in *robo4 *siRNA transfected cells, we have performed biochemical-binding analysis taking advantage of AP-tag on Slit2. AP-Slit2N was incubated with control *lacZ *or *robo4 *siRNA transfected endothelial cells, and bound Slit2 was detected by AP activity in the lysate. AP activity was higher in control *lacZ *siRNA than *robo4 *siRNA transfected endothelial cells suggesting that the presence of Robo4 facilitates Slit2 interaction on endothelial cells (Fig. 3B). Slit2 also interacts with Robo1 and this interaction occurs via the immunoglobulin (IgG) domains in Robo1 and the second leucine rich repeat sequence in Slit2 [19]. The K_d _of the Slit2-Robo1 interaction is reported to be 8–10 nM [19,26] and since the IgG domain is conserved in Robo1 and Robo4, we predict similar K_d_'s for Slit2-Robo4 interaction. The migration and biochemical binding analysis data taken together suggest that Slit2 binds to endothelial cells expressing Robo1 and Robo4 and inhibits their migration.

### Slit2 shows both chemotactic and chemokinetic effects on endothelial cells and the chemotactic response is in part through Robo4

*Robo4 *siRNA transfected cells did not respond to serum (Figs. 2A and 2B) but, remarkably, show a three-fold increase in migration to a Slit2 gradient when compared to control *lacZ *siRNA cells (Fig. 3A, black bars). Similar results are observed in mouse *robo4 *knockout cells. Mouse *robo4 *knockout endothelial cells migrate to VEGF-A better than control cells [7]. These results argue that irrespective of the stimulus, VEGF or Slit2, *robo4 *siRNA or *robo4 *knockout endothelial cells show a pro-migratory response.

To investigate why *robo4 *knockdown cells migrate to a Slit2 gradient, we have determined whether the migration response was chemotactic or chemokinetic. Chemotaxis is referred to directional movement towards or away from a gradient while chemokinesis is a cellular response associated to speeding up or slowing down movement and is gradient independent [27]. To differentiate between the two types of migration, we have performed the Boyden chamber assay with control *lacZ *siRNA and *robo4 *siRNA transfected cells to serum or Slit2. Previous reports of Slit2 on leukocyte migration clearly showed that irrespective of where Slit2 was present, upper, lower or both chambers, Slit2 inhibited SDF-1α induced leukocyte migration arguing for a chemokinetic and chemotactic inhibitory activity for Slit2 in this system [28]. Therefore, we hypothesized that Slit2 behaves similarly in endothelial cell migration.

Slit2 in the upper, lower or both chambers showed similar increased motility of *robo4 *siRNA cells relative to that of control *lacZ *siRNA cells (Fig. 3C, Slit2 bars). Therefore, the positive response to Slit2 is chemokinetic. In contrast, serum stimulated only chemotactic motility of control cells. Chemotaxis to serum was blocked in *robo4 *siRNA cells (Fig. 3C, FBS bars), and this blockade was only detected when serum was in the bottom chamber. The serum response of *robo4 *knockdown cells therefore excludes the possibility that *robo4 *knockdown cells by themselves display random movement. The Slit response on the other hand is a combination of chemotaxis and chemokinesis. Therefore, our data indicate that Slit2 shows both chemotactic and chemokinetic effects on endothelial cells, and the chemotactic response is in part through Robo4 while serum mediates an exclusive chemotactic response on *robo4 *knockdown endothelial cells.

### Mechanism of Slit2 inhibition of endothelial migration is independent of the Rho GTPase pathway

Previously, we had shown that Rho GTPases were activated by Robo4 in endothelial cells [22]. To investigate whether active Rho GTPases are modulated by Slit2 treatment of endothelial cells, we have checked by pulldown assay for Cdc42-GTP levels in control endothelial cells. Slit2 was incubated for 5, 10 (data not shown) and 15 min with control endothelial cells and pulldown analysis was performed on lysates from the treated cells (Fig. 3D). We did not convincingly notice an up-regulation of Cdc42-GTP levels in endothelial cells treated with Slit2 in either 5 or 15 min incubation times. (Fig. 3D, compare lanes 1 & 3 with 2 & 4 respectively). Since Slit2 treated endothelial cells do not migrate and show no activation of Rho GTPases, the mechanism of Slit2 inhibition of endothelial migration is most likely independent of the Rho GTPase pathway. It is important to note that our data supports the conclusions of Park [6] and Seth [18]*et al*., that Slit2 inhibits migration of endothelial cells. However, removal of Robo4 alters the Slit2 response dramatically in that the cells move independent of the Slit gradient. This data can also be interpreted as the "removal of the brake" scenario where once the cellular brake is removed the cells begin to move randomly to Slit2 but not to serum suggesting the presence of an as yet unidentified ligand to Robo4 in serum that mediates a specific chemotactic positive response via Robo4. The mouse *robo4 *knockout endothelial cell when compared to mouse wild-type lung microvascular endothelial cell also show a similar pro-migratory response to VEGF-A [7] and therefore suggests a common chemokinetic response to single growth factors by *robo4 *loss-of-function endothelial cells.

### Vilse, a Cdc42-GAP interacts with Robo4 cytoplasmic tail

**Figure 4:**
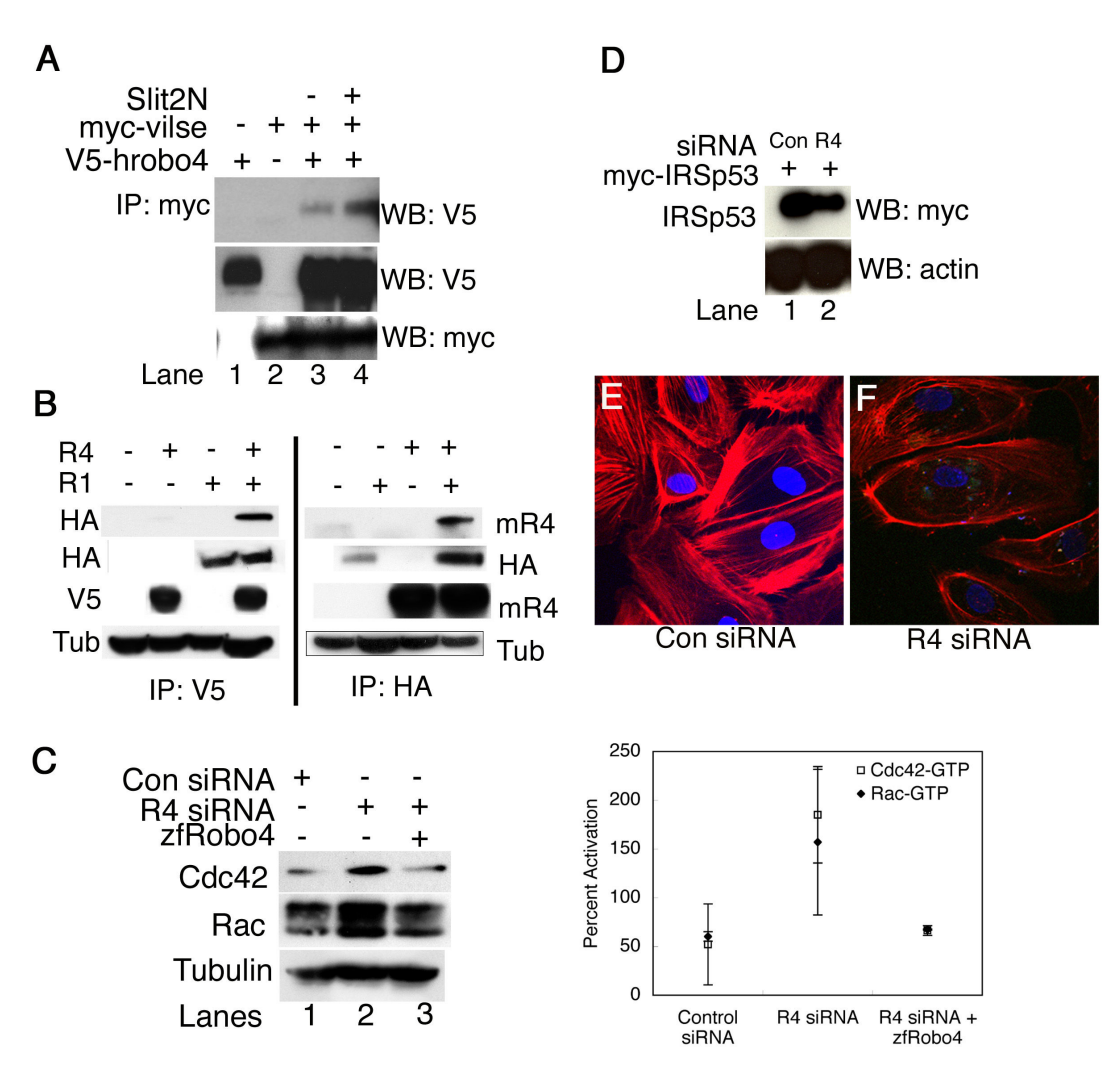
**Vilse and Robo1 interact with Robo4 and Rho GTPase homeostasis is important for Robo4 mediated directional migration**. **A **shows the interaction between Robo4 and Vilse in 293 cells. Myc-Vilse and V5-tagged human Robo4 were co-transfected into 293 cells in different combinations as indicated by + and -. Slit2N was added to the transfected cells and lysates were immunoprecipitated by myc antibody followed by western with V5 antibody. **B **shows IP/Western analysis for rat Robo1 and human Robo4 (left panel) or rat Robo1 and mouse Robo4 (right panel). The antibodies are indicated to the left and right of the gels. Tub: tubulin, mR4: mouse Robo4. The top gel in each panel represents the IP'd lysate western blotted with the indicated antibody. The rest of the gels are western blots for the respective proteins either present (+) or absent (-) as indicated for each sample in the top of the gel. **C **shows pull down analysis for Cdc42-GTP and Rac-GTP in Con (control) siRNA, *robo4 *(R4) siRNA and *robo4 *siRNA plus zfRobo4 transfected endothelial cells. Quantitation of the western blots was performed as described before [22]. Antibodies used for westerns are shown to the left of the blot. **D **shows western blots with myc and actin antibody of lysates from control *lacZ *siRNA (lane 1) or *robo4 *siRNA (lane 2) co-transfected with myc-IRSp53 constructs in endothelial cells. **E **and **F **are confocal images of F-actin phalloidin (red) stained endothelial cells transfected with reagents shown below the panel. Slides were mounted in media containing DAPI (blue), which stains nuclei. For **A, B, C **and **D **+ indicates addition of the reagent on the left, IP-immunoprecipitation, WB-western blot, antibodies indicated on right, For panels **C**, **D, E **and **F **Con: control *lacZ *siRNA, R4: *robo4 *siRNA.

Robo4 over-expressing endothelial cells show high levels of Cdc42-GTP and Rac-GTP [22] proteins. Whether the Cdc42-GTP up-regulation was a result of a guanine exchange factor (GEF) up-regulation or a GTPase activating protein (GAP) sequestering was not known. We made an educated guess on Vilse (Cdc42-GAP), since Vilse interacts with the intracellular CC2 domain in Robo1 that is shared by Robo4 as well [29]. Vilse indeed interacts with Robo4 cytoplasmic tail (Fig. 4A, lane 3) at least in HEK293T cells. Following Slit2 treatment, a slight increase in Robo4 pulldown levels is noticed (Fig. 4A, lane 4) but this increase is not observed in a consistent basis and therefore Vilse's interaction and its role in Slit2-Robo4 signaling is not clear. However, since serum was present in the pull-down assays, it is conceivable that Vilse interaction to Robo4 cytoplasmic tail is triggered by serum. Therefore, it is possible that serum mediated recruitment of Vilse, a Cdc42-GAP to Robo4 cytoplasmic tail is responsible for Rho GTPase activation. In terms of Slit2, we hypothesize that Rho GTPase independent mechanisms are in play for Slit2 inhibition of endothelial cell migration.

### Robo1 and Robo4 interact *in vitro*

Robo1 and Robo4 share two common features in that they bind Slit2 extracellularly and interact with Vilse intracellularly. Since Robos are known to homo- or hetero-dimerize [11], we investigated if Robo1 and Robo4 interact with each other by immunoprecipitation (IP) and western blot (Fig. 4B). In one set of experiments, we transfected HA-tagged rat Robo1 and V5-tagged human Robo4 (Fig. 4B, left panel), while in a second set of experiments, we transfected HA-tagged rat Robo1 construct and mouse Robo4 constructs (Fig. 4B, right panel) into HEK293T cells either alone or together. IP with V5 antibody (Fig. 4B, IP: V5, left panel) brought down Robo1 (Fig. 4B, left panel, last lane, top HA blot). Conversely, IP with HA antibody (Fig. 4B, IP: HA, right panel) resulted in pull down of mouse Robo4 (Fig. 4B, right panel, last lane, top mR4 blot). This data suggests that both mouse and human Robo4 interact with rat Robo1 and that similar to neuronal Robos, vascular Robos also interact.

### Rho GTPase homeostasis is essential for Robo4-induced migration

To investigate the active Cdc42 and Rac levels in *robo4 *knockdown cells, we have performed biochemical pull down analysis for Cdc42-GTP and Rac-GTP in the *robo4 *knockdown endothelial cells. Unexpectedly, the knockdown cells showed increased levels of Cdc42 and Rac-GTP (Fig. 4C, lane 2), which return to baseline in cells transfected with zebrafish Robo4 (Fig. 4C, lane 3). When combined with migration rescue experiment (Fig. 2B, R4siRNA + zfRobo4 black bar), this result suggests that restoring the balance of active vs. inactive Rho GTPases in an endothelial cell is required for chemotaxis. It is also worth noting that the previously published pull down data showed lower amounts of active Cdc42 and Rac in *robo4 *knockdown embryos [22], which was performed from whole embryo lysates and not from endothelial cells in the embryo.

Although *robo4 *knockdown endothelial cells contain high levels of Cdc42-GTP (Fig. 4C, lane 2), they do not migrate to serum (Fig. 2A). This result suggests that Cdc42-GTP level alone is not enough for directional migration but the interaction of active Cdc42 presumably with other proteins involved in filopodia formation or migration is important for this function. One such protein previously implicated in Cdc42-GTP induced filopodia formation is insulin receptor substrate protein 53 (IRSp53), a SH3 domain-containing adaptor molecule. IRSp53 protein interacts with Cdc42-GTP to promote filopodia formation in COS or Swiss 3T3 cells via an IRSp53 effector protein Mena, an Ena/VASP family member [30]. It is known that interaction of Cdc42 with the CRIB motif of IRSp53 relieves an intramolecular autoinhibitory interaction with the N terminus, allowing the recruitment of Mena to the IRSp53 SH3 domain [30]. The IRSp53:Mena complex initiates actin filament assembly into filopodia. Since Robo4 interacts with Mena [6], we asked whether IRSp53 protein, a target of active Cdc42 was involved in Robo4 signaling in endothelial cells. In *robo4 *knockdown endothelial cells co-transfected with myc-IRSp53 construct (Fig. 4D, lane R4), we noticed that the IRSp53 protein levels were lower when compared to samples of control *lacZ *siRNA transfected cells with myc-IRSp53 (Fig. 4D, lane Con). We have stained for F-actin phalloidin in *robo4 *siRNA (Fig. 4E) and control *lacZ *siRNA (Fig. 4F) cells and clearly noticed a loss of organized actin stress fibers in *robo4 *siRNA transfected cells. So, taking the IRSp53 stability (Fig. 4D), actin phalloidin staining (Figs. 4E and 4F) and activated Rho GTPase in *robo4 *knockdown cell (Fig. 4C) data together, a strong correlative argument can be made that in the absence of Robo4, IRSp53 is unavailable for active Cdc42-GTP interaction resulting in blocking of downstream events that lead to actin nucleation and filopodia formation. Further, the zebrafish Robo4 co-transfected sample (Fig. 4C, lane 3) shows Rho GTPase levels comparable to baseline thus allowing for endothelial cell chemotaxis to serum (Fig. 2B). This data also suggests that stabilization of IRSp53 protein may allow for excess Cdc42-GTP to be used for actin nucleation, and that a one to one correlation between IRSp53 and Cdc42-GTP molecules in a given cell may dictate the actin nucleation and filopodia formation process.

### Proposed mechanism of Robo4 function in endothelial cell migration

**Figure 5:**
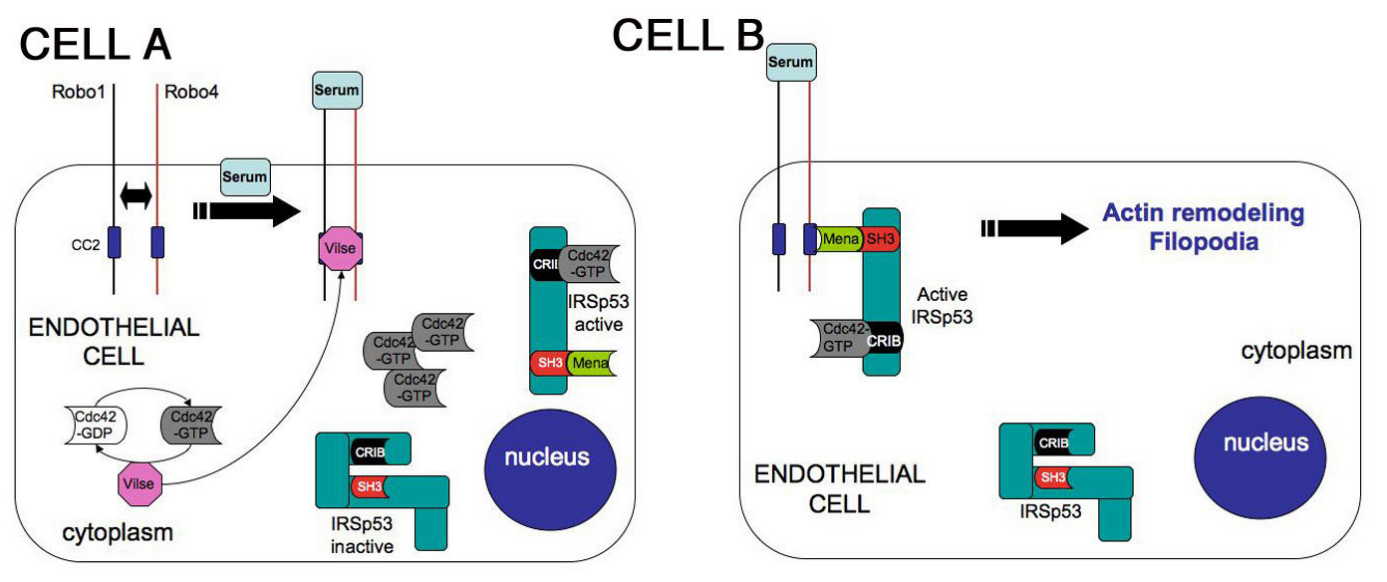
**Model depicting Robo1 and Robo4 mechanism of action in endothelial cell guidance**. **Cell A**: In resting state, in the absence of ligand (serum or Slit2), Robo1 and Robo4 interact and stay in an inactive conformation. On ligand binding, Vilse, a Cdc42-GAP is recruited to CC2 domain of Robo1 and Robo4 resulting in an increase in intracellular Cdc42-GTP levels. The Cdc42-GTP binds to CRIB domain in IRSp53, which changes conformation of IRSp53 from inactive to active state. The active conformation of IRSp53 allows Mena to interact with the exposed SH3 domain of IRSp53. **Cell B**: In an activated state, Mena recruits the complex to CC2 domain of Robo4's cytoplasmic tail or directly mediates actin nucleation resulting in filopodia formation and directional migration. This cartoon is a working model and is not conclusive.

Taking all the results in this study together, we propose a working model (Fig. 5) that is built on previous models [10]. In this model, we will integrate the following molecules: Robo1, Robo4, Slit2, Non-Slit ligand, Cdc42-GTP, IRSp53, Vilse and Mena. Here, two assumptions are in place: (1) Slit mediated migration is chemotactic and chemokinetic in nature while serum (non-Slit) mediated migration is exclusively chemotactic in nature, (2) Signaling outputs of ligand-receptor complex is context dependent and active switching between Robo1 and Robo4 at cell surface dictates directional migration of endothelial cell.

In situation when chemokinesis and chemotaxis are required concomitantly such as wound healing, the Slit pathway is active. In the resting state i.e., in the absence of Slit, cell surface Robos physically interact with each other, which may keep both Robos in an inactive state. Another possibility is that only one Robo is expressed at any given time on the cell surface and acts singularly since we know that Robo levels on the cell surface are tightly regulated. Therefore, the physical interaction is a safety mechanism of aberrantly activating Robos on cell surface. Slit binds to Robo1 and Robo4, and it is not clear if this interaction is dependent on the presence of the other Robo. Two consequences can be predicted: (a) a direct negative stimulus emanates from Robo1 or (b) the positive stimulus from Robo4 is blocked. Both the predictions involve a Rho GTPase independent mechanism in down stream signaling events. The net output is migration of the endothelial cell forward or away from the target site.

In situation where you require exclusive chemotaxis such as during development of ISVs during embryonic development, a non-Slit pathway is active. Again, in the resting state Robo cell surface levels are tightly regulated by one of three ways: (1) physical interaction between the two Robos or, (2) one Robo is selectively down-regulated or, (3) only the particular Robo of relevance is expressed on the cell of interest in this case the tip cell. To continue a directional path for the tip cell such as along the chevron of the somite, a non-Slit ligand binds to Robo4 resulting in recruitment of Vilse, the net result is activation of Cdc42. The activated Cdc42 interacts with the stabilized IRSp53 in the endothelial tip cell allowing the SH3 domain of IRSp53 to interact with Mena (Fig. 5). This complex of Cdc42-GTP-IRSp53-Mena results in actin cytoskeleton rearrangement leading to filopodia formation and migration.

### Robo4 function in tip and stalk cells

Robos were originally identified as axon guidance molecules and therefore a vascular Robo, Robo1 or Robo4 was expected to function in a growth cone analogous structure in endothelial cells namely tip cells. The retinal vascular bed has been shown to contain two distinct cell types in the developing vasculature namely the tip cell and the following stalk cell [31]. In the retinal vasculature, it was shown that tip cells do not proliferate but merely guide the following proliferative stalk cell by a VEGF-dependant process. The combined tip and stalk cell activity is thought to extend the developing endothelial sprouts. Recent evidence in zebrafish has challenged the notion that tip cells don't proliferate [32] since zebrafish trunk ISV tip cells do. This result argues that tip cells function is tissue dependant. In zebrafish, we have noted that *robo4 *is expressed along the entire length of the intersomitic sprout comprising of both the tip and stalk cell [5], and functions in a manner that is anticipated for axon guidance molecules in that *robo4 *knockdown embryos display ISV defects. Whether proliferation of ISV cells has altered in *robo4 *knockdown embryos is not known and is being investigated. Recent result of *robo4 *expression in mouse stalk cell [7] raises the issue whether higher vertebrates, in particular mammals have adapted Robos for other functions besides guidance. This is particularly possible given the fact that *Robo4*^*AP*/*AP *^mouse did not show developmental vascular defects in the ISVs or cephalic vessels [7]. Other reasons include, Robo4 stalk and tip cell function are species specific, vascular permeability is different in mouse and zebrafish. In the *Robo4*^*AP*/*AP *^mouse it was shown that Slit2-Robo4 signaling intercepted the VEGF permeability-signaling axis and is conceivable that the Robo4-stalk cell-permeability function is Slit dependant while the Robo4-tip cell-guidance function is non-Slit dependant. The *in vivo *expression pattern of Slits in zebrafish [33,34] and our data in this study that shows serum mediates positive chemotactic response via Robo4 provides supporting evidence for the latter.

This study raises numerous questions. What mechanisms are in play for a cell to recognize when to invoke chemotaxis vs. chemokinesis pathways? The fine details of the model proposed here (Fig. 5) raise several questions. Is Mena released from Robo4 cytoplasmic tail on ligand binding? What is the ligand that mediates this action? What is the order of Mena and Vilse recruitment to the Robo4 cytoplasmic tail? Does IRSp53 protein stability control the levels of active Cdc42-GTP in endothelial cells? How do these complexes promote filopodia formation in endothelial cells? These and other issues form the basis of future work in the laboratory.

## Conclusion

Our data suggests that endothelial cells utilize Robos in a context dependant manner and signals from surrounding milieu [Serum (non-Slit) vs. Slit2] dictate different responses and perhaps signals in endothelial cells.

## Methods

### RNA isolation, RT-PCR, Real Time PCR and Reagents

Total RNA was isolated from HUVECs 48 h post transfection by the Trizol method. RT-PCR was performed by using primer pairs described previously for hRobo1 [8]; β-actin [8], and hRobo4 [18]. Real time PCR Primers for hRobo1: F: CCTTCCACCAGCAAAGACTC, R: TGAGGAACTGGGATCTCTGG; hRobo4: F: GCAGTCACTGGTGCTGGAG; R: GACCATGCTCACTGGGTTCT; and β-actin: F: GGCATCCTCACCCTGAAGTA, R: AGGTGTGGTGCCAGATTTTC. The vector pAP-Slit2N was obtained from Dr. Alain Chédotal (Université Paris). Myc-IRSp53 and myc-Vilse constructs were gifts from Dr. Alan Hall (Sloan Kettering Institute) and Dr. Greg Bashaw (University of Pennsylvania) respectively. Mouse Robo4 construct and mouse Robo4 antibody were gifts from Dr. Dean Y Li (University of Utah).

### Dicer RNA knockdown

The human *robo4 *region corresponding from 1324 bp to 1732 bp was amplified using primers F: GCTGCAGTCACTGGTGCTGGAGCTGG and R: GGTCCCGGGCATCCGCCCCCAGCCG. The human *robo1 *region corresponding from 127 bp to 579 bp was amplified using primers F: TAATACGACTCACTATAGGGCCAATCCCCACCTCTGATAA and R: ATTAACCCTCACTAAAGGGAGGTTGGCATTCCATTACTGC. SiRNA was generated according to BLOCK-iTTM Complete Dicer RNAi Kit (Invitrogen) recommendations. HUVEC cells were grown to 80% confluence and transfected with 125, 250, 750 and 1000 ng of siRNA per well by LipofectamineTM 2000 Reagent. For *robo1 *siRNA experiments, 250 and 500 ng were used and for control *lacZ *siRNA comparable doses were used. For rescue experiments, zebrafish Robo4 described previously [22] was used. We performed endothelial cell transfection with either the Lipofectamine 2000 reagent or the Magnetofection method.

### Rho GTPase pull down and Western Blot assays

Pull down assays were performed as previously described [22] with a modification that endothelial lysates were generated 24 h post transfection. For Fig. 2D, HUVECs were serum starved for 12 h prior to treatment with AP-Slit2N (40 ng/ml) for the indicated times. Lysates were generated from treated samples using buffer provided by the Pierce Cdc42 pull down assay kit. For determining Robo4 protein levels of knockdown experiments (Fig. 1E), western blots were performed on lysates from co-transfected samples containing V5-tag human Robo4 and control or *robo4 *siRNA. Lysates were also probed for actin (Sigma). For co-IP experiments in HEK293T cells, equal amounts of the two plasmids (5 μg) were either co-transfected or not depending on the combination desired for 36 h. Lysates were made in RIPA buffer (Sigma) and pre-cleared with protein G-agarose (Pierce) for 3 h. Precleared lysates were IP'd with myc antibody (1:1000) (Cell Signaling) for overnight. The myc antibody was captured by protein G-agarose for 3 h. All IP steps were performed at 4°C. The beads were washed three times with RIPA buffer and 2× sample buffer was added prior to boiling. The heated samples were resolved on 7% SDS-PAGE (Novex) and western blot was performed as described previously [22]. In case of Slit2 treated samples, 40 ng/ml of AP-Slit2N was incubated with co-transfected cells for 1 h. Five micrograms of myc-tagged IRSp53 was co-transfected into *robo4 *siRNA (250 ng) or control *lacZ *siRNA (250 ng) endothelial cells by Lipofectamine 2000 reagent. Twenty-four hours post transfection lysates were made as described earlier. Quantitation of western blots were performed as described before [22].

### AP fusion protein generation, AP activity assays

For AP fusion proteins, HEK293T cells were transfected with pAP-tag5 and pAP-Slit2N constructs. Forty-eight hours post transfection; supernatant was collected by centrifugation to remove cell debris. All assays with AP proteins used HEK293T supernatants directly without further purification. AP controls processed in an identical manner were used as controls. AP binding assay was performed by incubation of equal amounts of AP and AP-Slit2N proteins (25 ng/mL) with control or *robo4 *siRNA cells for 1 h at 4°C. Cells were washed three time with 1× PBS and lysates were made with AP lysis buffer (GenHunter). The lysates were heat inactivated at 65°C for 10 min to inactivate background phosphatase activity and protein concentration was measured using bicinchoninic acid (BCA) assay (Pierce). Equal amounts of protein were used for detecting AP activity. AP activity was measured by adding AP assay reagent A (GenHunter) and absorbance read at 405 nm using a microplate reader.

### Migration assay

Migration assay was performed as described before [22]. All migration assays were performed with HUVECs passage 2–4. Briefly, 20,000 cells from each experimental group were placed in the upper chamber in a final volume of 350 μL of serum free medium. Serum (10% FBS) or VEGF-A (100 ng/mL in serum free medium) was placed in the bottom chamber in a final volume of 500 μL. All migration assays were conducted for 5 h at 37°C and at the end of the assay the cells were fixed and counted as per the manufacturer's instructions (Dade Behring). For differentiating between chemokinesis and chemotaxis, a checkerboard format of adding Slit2 or serum to upper, lower or both chambers was performed.

### Confocal immunofluorescent microscopy

HUVEC cells were grown on cover slips, fixed with 4% formaldehyde in PBS for 1 h at room temperature, and permeabilized for 30 min with 0.1% Triton X-100 in PBS containing 1% BSA. Cells were incubated for 20 min with phalloidin conjugated to Texas Red (Molecular Probes, Inc.), and cover slips were mounted in DAPI media for confocal imaging using a Zeiss LSM510 confocal system mounted on a Zeiss Axiovert 100 M microscope.

## Authors' contributions

SK, GVS, PWL, KP and MP performed experiment, designed experiments and interpreted results. DR suggested experiments, interpreted results and edited the paper. RR co-ordinated the study, designed experiments and wrote the paper.
